# The combination of amlodipine/valsartan 5/160 mg produces less peripheral oedema than amlodipine 10 mg in hypertensive patients not adequately controlled with amlodipine 5 mg

**DOI:** 10.1111/j.1742-1241.2008.01977.x

**Published:** 2009-02

**Authors:** J Schrader, A Salvetti, C Calvo, E Akpinar, L Keeling, M Weisskopf, P Brunel

**Affiliations:** 1 St. Josef-HospitalCloppenburg, Germany; 2 Department of Internal Medicine, University of PisaPisa, Italy; 3 Hospital Clinico Universitario Santiago De Compostela, C/Choupana, s/nSpain; 4 Cukurova University Medical Faculty, Department of Family MedicineAdana, Turkey; 5 Clinical Development & Medical Affairs, Novartis Pharma AGBasel, Switzerland

## Abstract

**Aims:**

To demonstrate the benefit of the combination amlodipine/valsartan 5/160 mg over amlodipine 10 mg, in producing a lower incidence of peripheral oedema for a comparable mean sitting systolic blood pressure (MSSBP) reduction.

**Methods:**

After a 4-week amlodipine 5 mg run-in phase, inadequately controlled hypertension patients (aged ≥ 55 years, MSSBP ≥ 130 and ≤ 160 mmHg) were randomised to receive amlodipine/valsartan 5/160 mg or amlodipine 10 mg for 8 weeks, followed by amlodipine/valsartan 5/160 mg for 4 weeks for all patients. Primary variables were MSSBP change from baseline to week 8 and incidence of peripheral oedema reported as an AE. Resolution of peripheral oedema was assessed 4 weeks after switching patients from amlodipine 10 mg to amlodipine/ valsartan 5/160 mg.

**Results:**

At week 8, MSSBP showed greater reduction with amlodipine/valsartan 5/160 mg than amlodipine 10 mg (least square mean: −8.01 vs.−5.95 mmHg, p<0.001 for non-inferiority and p=0.002 for superiority). Systolic control, overall BP control and systolic response rate at week 8 were significantly higher with combination than amlodipine 10 mg (34 vs. 26%; 57 vs. 50%; 36.57 vs. 27.77%, respectively). Incidence of peripheral oedema was significantly lower with the combination than amlodipine 10 mg (6.6 vs. 31.1%, p<0.001). Peripheral oedema resolved in 56% patients who switched from amlodipine 10 mg to the combination, without the loss of effect on BP reduction.

**Conclusion:**

In non-responders to amlodipine 5 mg, treatment with amlodipine/valsartan 5/160 mg induced significantly less peripheral oedema than amlodipine 10 mg for similar BP reduction. Peripheral oedema resolved in > 50% patients switching from amlodipine 10 mg to the combination.

**What’s known**

Amlodipine, a widely used antihypertensive agent, causes peripheral oedema, an adverse event that is established to be dose-dependent, owing primarily to preferential arteriolar dilation. At the capillary level, venodilation should compensate for the arteriolar dilation and thus minimise amlodipine-induced peripheral oedema. The combination of valsartan and amlodipine is associated with better efficacy and tolerability than amlodipine alone.

**What’s new**

This study shows that adding valsartan to the common starting dose of amlodipine provides similar BP reduction with a better tolerability profile (leading to fewer discontinuations) when compared with the up-titration of amlodipine. This further enhances the clinical evidence for the early use of combination therapy, supported by effective BP reduction, improved tolerability and increased persistence on antihypertensive treatment.

## Introduction

Hypertension is a well-known major risk factor for target organ damage and cardiovascular (CV) clinical events (1), representing the number one underlying cause of death worldwide (2). Although available data from controlled clinical studies indicate that blood pressure (BP) reduction to target levels (BP < 140/90 mmHg in all hypertensive patients and < 130/80 mmHg in diabetic patients and high or very high-risk patients) is associated with a significant decrease in CV mortality and morbidity, such target BP values are difficult to achieve (1). Thus, available data indicate that a major challenge in the treatment of hypertension is inadequate BP control, with only 3–38% of patients worldwide (3) and 31–63% in developed countries attaining target BP of < 140/90 mmHg (4). Among various factors contributing to inadequate BP control, efficacy and tolerability of drug treatment seem to play a prominent role (1,3).

Monotherapy is a rational therapeutic approach in patients with mild BP elevation and low-to-moderate CV risk, with targeted BP < 140/90 mmHg (1). However, the ability of any agent used alone to achieve target BP values is limited to not more than 20–30% of hypertensive patients except in those with grade 1 hypertension (1). In responders to monotherapy, but with uncontrolled BP, the increase in dosage for drugs with dose-dependent efficacy constitutes a further rational therapeutic approach. But several antihypertensive drugs, including calcium antagonists, also have a dose-dependent tolerability profile. Therefore, increasing the dose could result in an increased incidence of adverse events (AEs), thus reducing patient compliance with therapy. In these patients, low-dose combination therapy is a better therapeutic alternative, provided that the combination includes drugs with different and complementary mechanisms of action, which could additionally potentiate the antihypertensive effect and improve tolerability while minimising individual side effects (1,5). Moreover, a fixed-dose combination of two drugs fulfilling the above mentioned criteria can simplify the treatment schedule and improve patient compliance (1,6).

Amlodipine, a dihydropyridine calcium channel blocker, is one of the most widely used agents in the treatment of hypertension (7) and is considered by many to be the most efficacious. However, a major hurdle with the use of amlodipine is the occurrence of peripheral oedema (8,9), when the dose is increased (10,11). Therefore, a rational therapeutic approach for preventing the peripheral oedema associated with amlodipine, would be to add a potent and highly selective blocker of the renin–angiotensin system (1), such as valsartan to compensate for the arteriolar dilation produced by amlodipine (12) by also dilating the venules (13), limiting fluid leakage into tissues. Thus, two key mechanisms are targeted to achieve rapid and optimal BP control (14) and indeed the combination of amlodipine and valsartan is associated with a significantly better BP-lowering effect and greater response rate compared with amlodipine alone (15).

In this study, we evaluated the non-inferiority of the combination amlodipine/valsartan 5/160 mg compared with amlodipine 10 mg with respect to antihypertensive efficacy, along with the incidence of peripheral oedema, in patients not adequately controlled with amlodipine 5 mg alone. We also assessed the incidence of peripheral oedema resolution when patients who developed peripheral oedema during 8 weeks of treatment were switched from amlodipine 10 mg to the combination of amlodipine/valsartan 5/160 mg.

## Methods

### Study design

This was a multi-centre, randomised, double-blind, double-dummy, placebo-controlled, parallel-group study evaluating the benefit of the amlodipine/valsartan 5/160 mg combination in reducing peripheral oedema, for the same BP lowering, compared with amlodipine 10 mg alone in patients with essential hypertension. The study was performed in 148 centres in 12 countries (Argentina, Chile, Ecuador, Finland, France, Germany, Italy, Norway, Spain, Sweden, Switzerland, Turkey) between January and November 2007 and was conducted in accordance with International Conference on Harmonization–Good Clinical Practice (ICH-GCP), Declaration of Helsinki and applicable local regulations. The study received approval from Institutional Review Board or Ethical Review Committee, and all patients provided written informed consent.

**Figure 1:**
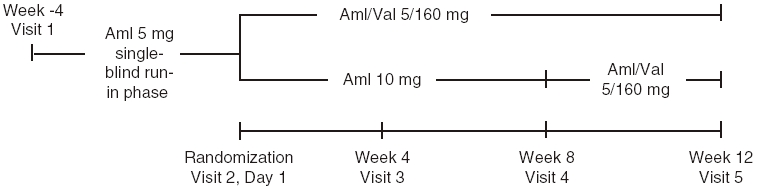
Schematic representation of the study design. Aml, amlodipine; Aml/Val, amlodipine/valsartan

The study comprised a 4-week, single-blind, amlodipine 5 mg run-in period and a 12-week, double-blind, active-treatment period. At the end of the single-blind, run-in period, patients whose mean sitting systolic BP (MSSBP) was not adequately controlled (MSSBP ≥ 130 and ≤ 160 mmHg) were randomised (1 : 1) to receive amlodipine/valsartan 5/160 mg combination or amlodipine 10 mg alone for 8 weeks. At week 8, patients who were on amlodipine/valsartan 5/160 mg combination continued the same treatment, whereas those who were on amlodipine 10 mg alone switched to the amlodipine/valsartan combination for an additional 4 weeks (Figure 1).

Patients were instructed to take the study medication orally with water once daily in the morning, regardless of meals, except on days of visit, when the medication was taken under the supervision of the investigator after BP assessment. On the first day of the double-blind phase, BP was measured in both arms using an Omron automated BP monitor, and the arm with higher diastolic BP reading was used at all subsequent visits. The MSSBP and mean sitting diastolic BP (MSDBP) were measured at trough three times (at 2-min intervals between measurements) at each visit, and the average of three readings was recorded for analysis.

### Study population

Male and female patients (aged ≥ 55 years) with essential systolic hypertension (MSSBP ≥ 140 and ≤ 160 mmHg, if not previously treated and MSSBP ≤ 160 mmHg, if pretreated on monotherapy) were enrolled into the run-in phase at week-4.

The inclusion criteria for randomisation were patients with MSSBP ≥ 130 and ≤ 160 mmHg and no peripheral oedema. Exclusion criteria were patients with MSSBP > 180 mmHg or MSDBP > 110 mmHg at any time during the single-blind run-in phase; patients on more than one antihypertensive agent on the day of randomisation; secondary hypertension; suspected contraindications; significant CV and cerebrovascular, renal, hepatic or pancreatic diseases; type 1 diabetes mellitus and inadequately controlled type 2 diabetes mellitus; any surgical or medical condition, which could affect drug disposition or place the patient at higher risk; and women of child-bearing potential.

### Efficacy

One of the co-primary variables was change in MSSBP from baseline (day of randomisation) up to week 8 [last observation carried forward (LOCF)]. The secondary efficacy variables were the (i) change in MSSBP and MSDBP, (ii) systolic control rate (defined as MSSBP < 130 mmHg), (iii) overall control rate (defined as BP < 140/90 mmHg for non-diabetic patients and < 130/80 mmHg for diabetic patients), and systolic response rate (defined as MSSBP < 130 mmHg or at least 20 mmHg reduction from baseline in MSSBP) at weeks 4, 8 and 12 in each treatment group.

### Safety and tolerability

#### Peripheral oedema

The second co-primary variable was the presence of peripheral oedema, which was evaluated at every visit. The evaluation was based on spontaneously reported oedema by the patients and on the presence of signs of oedema on physical examination of the patient by the investigator. Patients were counted as having peripheral oedema if it occurred at any time postdose, up to and including week 8. The absolute number and proportion of patients with peripheral oedema at any time postdose and up to and including week 8 were summarised by severity (none, mild, moderate and severe) and treatment group. If the severity changed over time, the maximum severity was used for analyses. If a patient experienced more than one occurrence of peripheral oedema between start of the double-blind phase and week 8, it was only counted once in the analysis. In the case of patients who discontinued before week 8, the information available up to that point was used for analysis.

The absolute number and proportion of patients with peripheral oedema resolution at week 12 (after switching from amlodipine 10 mg to the amlodipine/valsartan 5/160 mg combination at week 8 or continuing on amlodipine/valsartan 5/160 mg for 12 weeks) were also recorded.

#### Further safety assessments

Other safety assessments included regular monitoring and recording of all AEs, vital signs and physical examination, laboratory investigations and ECG as per the visit schedules. Each AE was described by its duration, severity and relationship to the study drug.

### Statistical analysis

All statistical analyses were performed using non-inferiority tests at the one-sided significance level of 0.025. A sample size of 916 patients (458 patients per group) was required (90% power) to show non-inferiority (change in MSSBP and MSDBP) between the treatment groups. The intent-to-treat population was used for the efficacy analyses. The safety population (defined as patients who received at least one dose of double-blind study drug) was used for the analysis of data on peripheral oedema and AEs.

For the primary efficacy variable, the last postbaseline MSSBP measurement collected (LOCF) was used for the analysis in case of patients who discontinued prior to week 8. The change from baseline (day 1) in MSSBP and MSDBP at weeks 4, 8 and 12 was analysed using analysis of covariance (ANCOVA) with baseline as covariate and a non-inferiority margin of 3 mmHg for MSSBP and 2 mmHg for MSDBP. A logistic regression model was used for the analysis of the number of patients with systolic BP control and systolic response rate (with baseline MSSBP as covariate) and overall BP control (with baseline MSSBP and MSDBP as covariates). The proportion of patients who developed peripheral oedema in each treatment group up to and including week 8 was analysed using logistic regression, with treatment, region and diabetic status as fixed factors.

## Results

### Patient demographics

**Figure 2:**
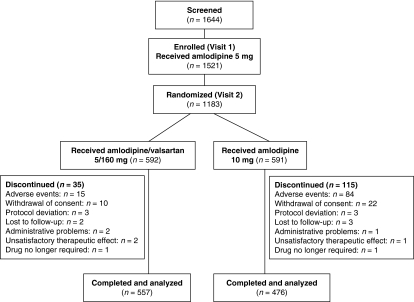
Overall patient disposition during the treatment period

Of 1644 patients who were screened, 1521 were enrolled into the single-blind amlodipine 5 mg, run-in phase (week-4). Overall, 1183 patients were randomised (1 : 1) to receive amlodipine/valsartan 5/160 mg (*n*=592) or amlodipine 10 mg (*n*=591) (Figure 2). Of these, 1033 patients completed the study, and the rate of completion was higher in the amlodipine/valsartan group (94.1%) than in the amlodipine 10 mg group (80.5%). The frequency of discontinuation caused by AEs was higher in the amlodipine group (14.2%) when compared with the amlodipine/valsartan group (2.5%).

Males and females were equally represented with an overall mean age of 65.5 years, and in both treatment groups most of the patients were Caucasian (95%). At baseline, after 4 weeks of treatment with amlodipine 5 mg, the overall MSSBP and MSDBP were 143.9 and 83.8 mmHg, respectively. The treatment groups were well-matched with respect to age, gender, race, body mass index, MSSBP, MSDBP, diabetes status and a history of prior antihypertensive medication (Table 1).

**Table 1:** Demographic and baseline characteristics of the patient population at randomisation

	Amlodipine/valsartan 5/160 mg *n*=592	Amlodipine 10 mg *n*=591	Total *n*=1183
Age (years) – [Mean (SD)]	65.6 (7.56)	65.4 (7.16)	65.5 (7.36)
**Gender –*n* (%)**			
Male	307 (51.9%)	307 (51.9%)	614 (51.9%)
Female	285 (48.1%)	284 (48.1%)	569 (48.1%)
**Race –*n* (%)**			
Caucasian	559 (94.4%)	560 (94.8%)	1119 (94.6%)
Other	33 (5.6%)	28 (4.7%)	61 (5.2%)
Asian	0	2 (0.3%)	2 (0.2%)
Pacific islander	0	1 (0.2%)	1 (0.1%)
BMI – kg/m^2^	28.9 (4.59)	28.7 (4.41)	28.8 (4.50)
MSSBP (mmHg)	143.4 (7.99)	144.4 (8.16)	143.9 (8.09)
MSDBP (mmHg)	83.3 (8.74)	84.2 (8.44)	83.8 (8.60)
**Diabetes status –*n* (%)**			
No	486 (82.1%)	493 (83.4%)	979 (82.8%)
Yes	106 (17.9%)	98 (16.6%)	204 (17.2%)
**Prior antihypertensive medication –*n* (%)**			
No	146 (24.7%)	120 (20.3%)	266 (22.5%)
Yes	446 (75.3%)	471 (79.7%)	917 (77.5%)

MSSBP, mean sitting systolic blood pressure; MSDBP, mean sitting diastolic blood pressure; BMI, body mass index.

### Efficacy

For change in MSSBP from baseline at week 8 (LOCF), the least square mean (LSM) reduction with the amlodipine/valsartan group was shown to be statistically non-inferior when compared with the amlodipine group [−8.01 vs.−5.95; 95% CI (−3.34, −0.79); p<0.001 for non-inferiority and p=0.002 for superiority].

**Figure 3:**
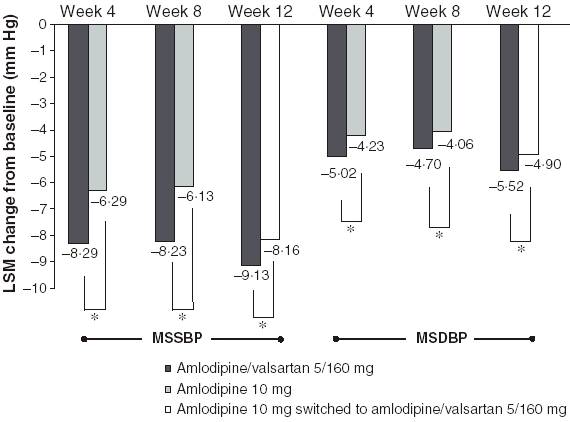
Change from baseline in MSSBP and MSDBP. *p < 0.001 for non-inferiority, between treatment groups. LSM, least square mean; MSSBP, mean sitting systolic blood pressure; MSDBP, mean sitting diastolic blood pressure

Non-inferiority was also observed with LSM reductions from baseline at week 4 (−8.29 vs. −6.29; p<0.001) and week 8 (−8.23 vs.−6.13; p<0.001) in MSSBP, at week 4 (−5.02 vs.−4.23; p<0.001) and week 8 (−4.70 vs.−4.06; p<0.001) in MSDBP and at week 12 after the switch from amlodipine 10 mg to amlodipine/valsartan 5/160 mg (−9.13 vs.−8.16; p<0.001 for MSSBP and −5.52 vs.−4.90; p<0.001 for MSDBP) between the amlodipine/valsartan and amlodipine treatment strategy groups (Figure 3).

The systolic control produced by the combination of amlodipine/valsartan was better than amlodipine alone, at week 4 [34.98 vs. 24.83%; 95% CI (1.22, 2.14); p<0.001] and week 8 [34.28 vs. 26.21%; 95% CI (1.06, 1.88); p=0.019], and similar after the switch from amlodipine 10 mg to amlodipine/valsartan 5/160 mg at week 12 [38.04 vs. 31.81%; 95% CI (0.92, 1.62); p=0.162].

**Figure 4:**
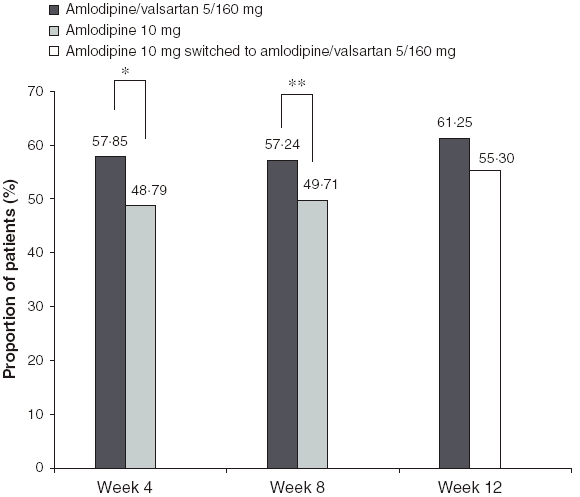
Effect of the treatment regimen on overall blood pressure (BP) control. *p < 0.001 between the treatment groups at week 4. **p = 0.019 between the treatment groups at week 8. Overall BP control is defined as BP < 140/90 mmHg for non-diabetic and < 130/80 mmHg for diabetic patients

Overall BP control was attained in a significantly higher proportion of patients when treated with the amlodipine/valsartan combination therapy than with amlodipine monotherapy at weeks 4 and 8 (Figure 4).

The systolic BP response rate was higher with amlodipine/valsartan 5/160 mg than with amlodipine 10 mg at week 4 [37.20 vs. 26.72, 95% CI (1.23, 2.08); p<0.001] and week 8 [36.57 vs. 27.77%, 95% CI (1.10, 1.90); p=0.009], and similar after the switch from amlodipine 10 mg to amlodipine/valsartan 5/160 mg at week 12 [40.36 vs. 35.76%; 95% CI (0.87, 1.48); p=0.347].

### Safety and tolerability

#### Peripheral oedema

The most frequently reported AE during the study was peripheral oedema, which was considerably higher in the amlodipine 10 mg group (31.5%) than in the amlodipine/valsartan 5/160 mg group (7.3%). The severity of peripheral oedema was also higher in the amlodipine 10 mg group compared with the amlodipine/valsartan 5/160 mg group (mild: 17.6 vs. 5.9%; moderate: 10.3 vs. 1.2%; severe: 3.6 vs. 0.2%, respectively).

**Figure 5:**
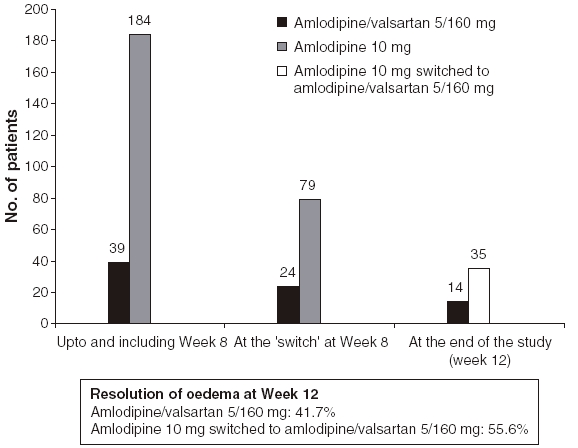
Incidence of peripheral oedema at week 8 and 12

The combination of amlodipine/valsartan 5/160 mg induced significantly less peripheral oedema than amlodipine 10 mg [6.6 vs. 31.1%; 96% CI (0.11, 0.22); p<0.001] up to and including week 8 (Table 2). The incidence of peripheral oedema decreased in both treatment groups from week 8 to 12. This decrease, however, was more pronounced in the group that was switched over from amlodipine 10 mg to amlodipine/valsartan 5/160 mg combination (31.1% at week 8 and 14.2% at week 12) than in the group that continued on amlodipine/valsartan up to week 12 (6.6% at week 8 and 4.9% at week 12) (Figure 5).

**Table 2:** Incidence and resolution of peripheral oedema

	Amlodipine/ valsartan 5/160 mg *n* (%)	Amlodipine 10 mg *n* (%)	Amlodipine switched to amlodipine/valsartan at week 8 *n* (%)
No. of patients randomised	592	591	–
No. of patients with peripheral oedema during the first 8 weeks	39 (6.6)	184 (31.1)*	–
Peripheral oedema suspected to be drug-related by physician	36 (6.1)	171 (28.9)	–
No. of patients treated after week 8 (switch**)	562	–	484
No. of patients with peripheral oedema entering switch phase	24 (4.3)	–	79 (16.3)
No. of patients with peripheral oedema resolution during switch phase	10 (1.8)	–	44 (9.1)
No. of patients with unresolved peripheral oedema during the switch phase	14 (2.5)	–	35 (7.2)
New cases of peripheral oedema during the switch phase	5 (0.9)	–	5 (1.0)

* p<0.001, between the two groups.

** At week 8, amlodipine 10 mg patients were switched to amlodipine/valsartan 5/160 mg; patients on amlodipine/valsartan 5/160 mg were continued on the same treatment regimen.

Resolution of peripheral oedema was observed in more than half of the patients (44 out of 79, excluding five new cases after week 8) after they were switched over from amlodipine 10 mg to the amlodipine/valsartan 5/160 mg combination (Table 2). Resolution of peripheral oedema at week 12 was higher in the patients who had switched from amlodipine 10 mg to amlodipine/valsartan 5/160 mg (55.6%) than in patients who were on the amlodipine/valsartan combination (41.7%).

#### Further safety results

Both treatment regimens were well tolerated. The overall incidence of AEs was higher in the amlodipine group (55.7%) than the amlodipine/valsartan group (43.9%). After peripheral oedema, the second most frequently reported AE related to the drug was headache, which also occurred at a higher frequency in the amlodipine group (3.2%) than in the amlodipine/valsartan group (2.2%). The frequencies of other AEs were comparable in both the groups.

The main reason for discontinuation was AEs (2.5% with amlodipine/valsartan vs. 14.2% in amlodipine) and the most frequently reported AE leading to discontinuation was peripheral oedema (1% with amlodipine/valsartan vs. 11.5% with amlodipine), followed by headache (0.2% with amlodipine/valsartan vs. 0.5% with amlodipine).

There were no deaths in this study, and serious adverse experiences (SAEs) were rare. Eleven patients (1.9%) in the amlodipine/valsartan group and eight patients (1.4%) in the amlodipine group experienced SAEs, which included two cases of serious cholecystitis in the amlodipine/valsartan group and one case each of gastrointestinal necrosis, gastrointestinal haemorrhage and inguinal hernia in the amlodipine group. None of these SAEs was suspected to be study drug related. There were no clinically notable laboratory findings in this study.

## Discussion

Amlodipine has a well-documented BP-lowering efficacy; however, in non-responders to amlodipine monotherapy, up-titration is limited by dose-dependent adverse effects such as peripheral oedema (11). This study evaluated the antihypertensive effect and tolerability of the combination of amlodipine/valsartan 5/160 mg compared with amlodipine 10 mg, in non-responders to amlodipine 5 mg. Among those non-responders, a higher proportion achieved BP control with low-dose combination therapy compared with high-dose amlodipine monotherapy (16). Adding an ARB, such as valsartan, presents a feasible and safe therapeutic alternative to dose escalation in such patients. (17).

While efficacy is the prerequisite for optimal BP control, tolerability is an important determinant of adherence to therapy and attainment of long-term BP goals. The high incidence of peripheral oedema with amlodipine is because of potent arteriolar or precapillary dilation (without dilation in the venules or postcapillary circulation). The resultant extravasation of fluid into the surrounding tissue manifests as peripheral oedema (8). Addition of valsartan in the combination dilates venous capacitance vessels with consequent intracapillary pressure normalisation, thereby minimising exudation of fluid from the inter-capillary space, and counteracting oedema caused by amlodipine (14). In previous studies (15,18), the combination of amlodipine and valsartan demonstrated a lower incidence of oedema in patients randomised to the combination. However, in this study, much of the reduction in the incidence of oedema seen with the combination is likely because of the different doses of amlodipine in the two arms.

In those patients from the high-dose amlodipine group experiencing oedema, resolution occurred in more than half of the cases after switching to the combination. However, these data must be considered in light of the fact that significantly more patients discontinued from the amlodipine monotherapy arm during the first 8 weeks of the study, primarily caused by peripheral oedema. This may have introduced a selection bias where only those patients whose oedema was less severe continued into the second phase of the study and were switched to the combination. Thus, one might surmise that the effect would have been different and had more patients experiencing oedema in the amlodipine 10 mg arm completed the study.

Aside from the lower incidence and resolution of peripheral oedema, the overall incidence of AEs (oedema being the most frequent AE) was also lower with the amlodipine/valsartan 5/160 mg combination than with amlodipine 10 mg. This led to many more discontinuations because of AEs with amlodipine monotherapy than with the combination. In addition to the added advantage of a better tolerability profile for a comparable efficacy, the administration of fixed-dose combination regimen in hypertension is associated with fewer discontinuations and better compliance than administration of two agents separately (19). Lack of discontinuation or persistence to the treatment regimen is essential for hypertension control and best clinical outcome (20).

## Conclusion

In those hypertensive patients not adequately responding to amlodipine 5 mg monotherapy, the combination of amlodipine/valsartan 5/160 mg induces significantly lesser peripheral oedema for similar BP reductions, and a better safety and tolerability profile than amlodipine 10 mg. In addition, in patients experiencing oedema in the high-dose amlodipine monotherapy arm, more than half resolved after switching to amlodipine/valsartan 5/160 mg. Thus, an effective alternative to amlodipine dose escalation in patients would be a combination of low-dose amlodipine and valsartan, which has a better antihypertensive effect than high-dose amlodipine and a better tolerability profile.
